# The Use of Percutaneous Lumbar Fixation Screws for Bilateral Pedicle Fractures with an Associated Dislocation of a Lumbar Disc Prosthesis

**DOI:** 10.1155/2013/676017

**Published:** 2013-11-04

**Authors:** William D. Harrison, David J. Harrison

**Affiliations:** Princess Margaret Hospital, Windsor, Berkshire SL4 3SJ, UK

## Abstract

*Study Design.* Case report. *Objective.* To identify a safe technique for salvage surgery following complications of total disc replacement. *Summary of Background Data.* Lumbar total disc replacement (TDR) is considered by some as the gold standard for discogenic back pain. Revision techniques for TDR and their complications are in their infancy. This case describes a successful method of fixation for this complex presentation. *Methods and Results.* A 48-year-old male with lumbar degenerative disc disease and no comorbidities. Approximately two weeks postoperatively for a TDR, the patient represented with acute severe back pain and the TDR polyethylene inlay was identified as dislocated anteriorly. Subsequent revision surgery failed immediately as the polyethylene inlay redislocated intraoperatively. Further radiology identified bilateral pedicle fractures, previously unseen on the plain films. The salvage fusion of L5/S1 reutilized the anterior approach with an interbody fusion cage and bone graft. The patient was then turned intraoperatively and redraped. The percutaneous pedicle screws were used to fix L5 to the sacral body via the paracoccygeal corridor. *Conclusion.* The robust locking screw in the percutaneous screw allowed a complete fixation of the pedicle fractures. At 3-year followup, the patient has an excellent result and has returned to playing golf.

## 1. Introduction

Lumbar disc replacement is becoming a popular surgical choice in the management of discogenic back pain. At present, there are good short- and medium-term outcomes in patients receiving lumbar disc prostheses as opposed to the more traditional interbody vertebral fusion. Long-term outcomes are currently under the spotlight as the practice of modern disc replacement enters the third decade of usage. The indications for total disc replacement (TDR) remain specific and the procedure should only be done in valid cases of discogenic back pain in selected patients.

Charité III (LINK SB, DePuy, Warsaw, IN, USA) was the TDR of choice in much of America and Europe during the late 90s and onwards. The design is modular, comprising two metal endplate components fashioned with convex articulating surfaces which oppose a central polymer inlay component. Much of the long-term data for TDR has been generated from the largely successful Charité III design. Complications following TDR are poorly understood and remedies to salvage function following complications are in their infancy.

Short-term complications of TDR include disruption to vascular and neurological structures during the approach, retrograde ejaculation, and haematoma. In the last decade there have been a handful of unfortunate cases of anterior dislocation of the polyethylene inlay of Charité III and ProDisc-L (Synthes, USA) prostheses [1]. These cases have been identified as unusual and unforeseen complications. In one case of pedicle fracture with no dislocation conservative management was suitable [1]. In all of the polyethylene inlay dislocations there was no attempt to revise the TDR and an interbody fusion was required in each case [1–5]. The mechanism of dislocation has been attributed to an unusual bilateral pars fracture, usually in the L5 vertebra, which causes spondylolisthesis and instability of the prosthesis. The potential causes of pars defects are numerous, including congenital absence, failure to develop in childhood, stress fractures, osteoporotic bone, and occasionally from extrinsic or iatrogenic trauma such as instrumentation during TDR placement. Maurer et al. describe a clear case of bilateral pedicle fracture following a microdiscectomy, a procedure that the patient in our case underwent [6].

The TDR related pars fractures have occurred during rehabilitation, with a presumed iatrogenic stress on the prosthetic endplate predisposing the pars and pedicle to fracture. One paper hypothesized that this was due to the inbuilt lordosis of the implant causing excessive stress on the core insertion [4]. This may be further exaggerated at the L5/S1 level which is thought to possess 40% of the inbuilt lumbar lordosis in the normal spine [7]. It is possible that increasing prosthetic lordosis secondary to implant subsidence over time may cause an increasing frequency of these complex pars fractures. 

Regardless of the mechanism of injury, a dislocated TDR with bilateral pedicle fractures poses a significant issue. Long-term complications of TDR and the subsequent management strategies are not well described.

This case demonstrates the success of using a percutaneous lumbar fixation system (PERPOS-PLS) from Interventional Spine (Irvine, CA, USA) to salvage a significant complication following TDR. The relative benefits of the technique and use of the system are described in this paper.

## 2. Case History

A 48-year-old male recruitment agent had suffered from mechanical lumbar back pain since the age of thirty. This patient had no other comorbidities and enjoyed playing regular golf and the occasional game of rugby.

The back pain had been manageable until an acute exacerbation occured following a posterior collision whilst being in his car, characterized by severe pain radiating down his right leg. There was no motor or sensory deficit.

Attendance at a neurosurgical spinal clinic confirmed degenerative disc disease in L4/5 and L5/S1, which was successfully managed with an L5/S1 microdiscectomy. A few years after the microdiscectomy, the back pain returned despite frequent epidural injections, core stability physiotherapy, and multimodal analgesia.

The patient was referred to spinal orthopaedic care for a review of ongoing symptoms and further management. Magnetic resonance imaging (MRI) confirmed degenerative disc disease in L4/5 with associated canal stenosis, L5/S1 foraminal stenosis at the lateral recess with an osteophytic discal bar, and an annular disc protrusion. A subsequent discogram of L4/5 demonstrated a slightly disorganised disc that was asymptomatic on pressurisation. The L5/S1 disc was sufficiently damaged to justify total lumbar disc replacement. 

**Figure 1:**
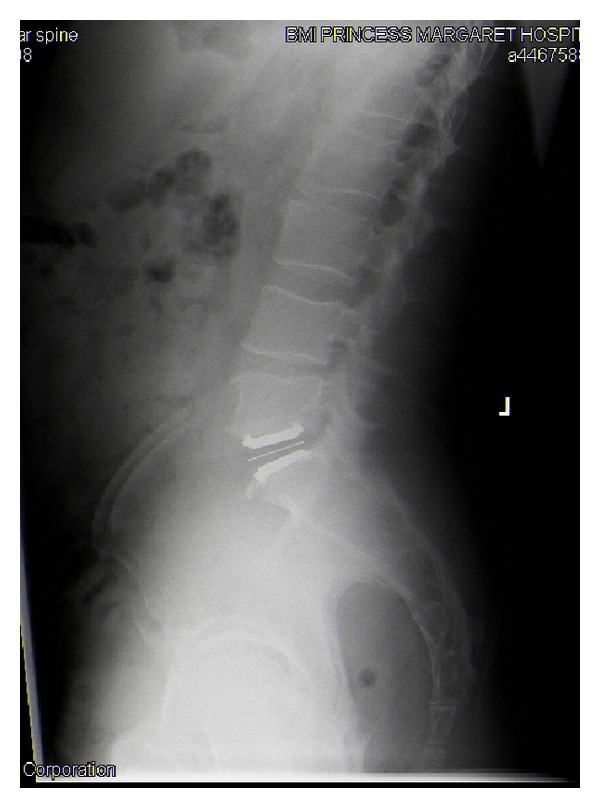
Plain films showing initial placement of total disc replacement.

**Figure 2:**
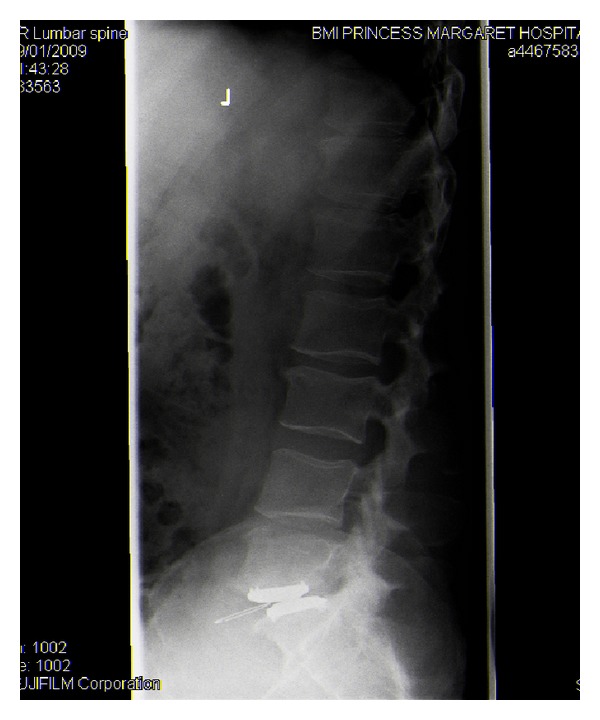
Plain radiograph showing a dislocated polyethylene inlay, but no pedicle fracture is seen.

A single level TDR at the L5/S1 disc space was performed as an elective procedure (Figure 1). The procedure was undertaken through a transverse abdominal incision with a transperitoneal approach. There were no recognised complications intraoperatively and the patient made a good initial recovery. Approximately 2 weeks postoperatively, the patient experienced extreme lumbar back pain and reduced range of movement following full spinal flexion. An emergency plain film X-ray identified an anterior dislocation of the polymer disc component (Figure 2).

**Figure 3:**
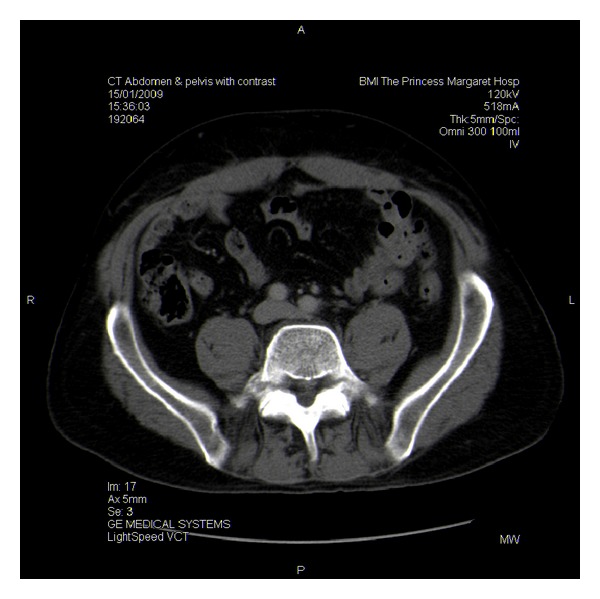
Axial view of bilateral L5 pedicle fracture.

**Figure 4:**
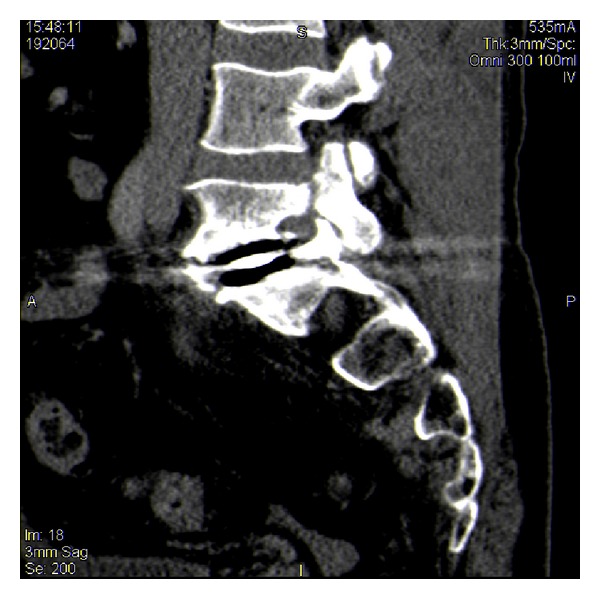
Sagittal view of L5 pedicle fracture.

A CT scan demonstrated the dislocated polymer disc abutting the left common iliac vein (but not the right common iliac vessels) with a small amount of inflammatory reaction and bilateral pars fractures with a grade 1 spondylolisthesis (Figures 3 and 4). Doppler blood flow to the distal lower extremities remained normal throughout the perioperative period.

It is unclear in this particular instance whether the pars defect was a missed preexisting defect following the previous microdiscectomy, acquired during surgical instrumentation, or a postoperative complication due to abnormal endplate forces during rehabilitation.

## 3. Operative Management

An attempt at relocation of the prosthesis resulted in redislocation intra-operatively, making a salvage fusion compulsory to achieve stability. On January 26, 2009, eleven weeks after the primary TDR, a salvage fusion was achieved using two approaches in sequence during the same anaesthetic.

The original anterior transperitoneal approach was reutilized with the patient in the supine extended Trendelenburg position to facilitate proximal gravitational migration of the abdominal contents.

**Figure 5:**
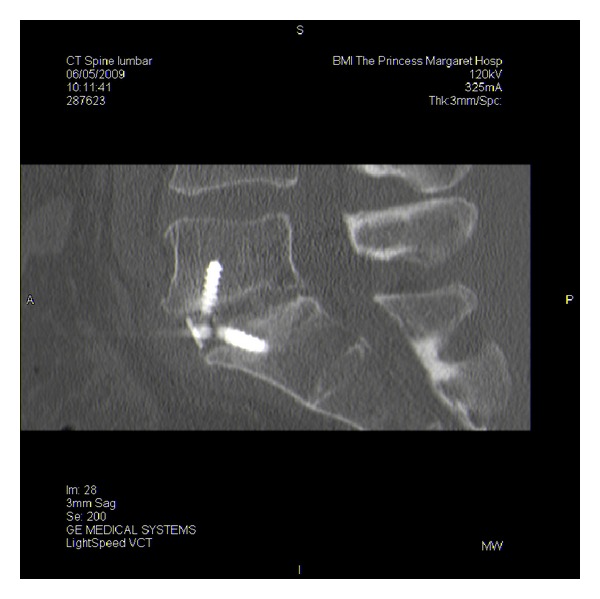
Anterior fusion of L5/S1 with intervertebral cage and bone graft.

It was possible to remove the TDR components by gently mobilizing the left common iliac vein that was crossing the front of the disc space. No vascular or retroperitoneal tissue trauma occurred and the anterior fusion was affected using a Stalif PEEK-Optima (Centinel Spine, Surgicraft, NY, USA). The interbody fusion cage was packed with artificial bone substituted securely and fixed with two titanium locking screws passed through preformed screw holes into the vertebral endplates (Figure 5).

Once the anterior approach had been closed, the patient was repositioned prone and the posterior surgery was performed using the percutaneous lumbar fixation screw (PERPOS-PLS). The original vertical median microsurgical discectomy scar was utilized. A very limited exposure using a Wiltse muscle-splitting approach provided access to the entry point for the pedicle on each side.

The slight disadvantage with the midline incision for a bilateral Wiltse muscle-splitting approach is that the incision has to be retracted strongly to about 4 cm off the midline on either side. If this had not been adequate, we would have been obliged to extend the midline incision or to make two additional paramedian incisions for a direct approach.

**Figure 6:**
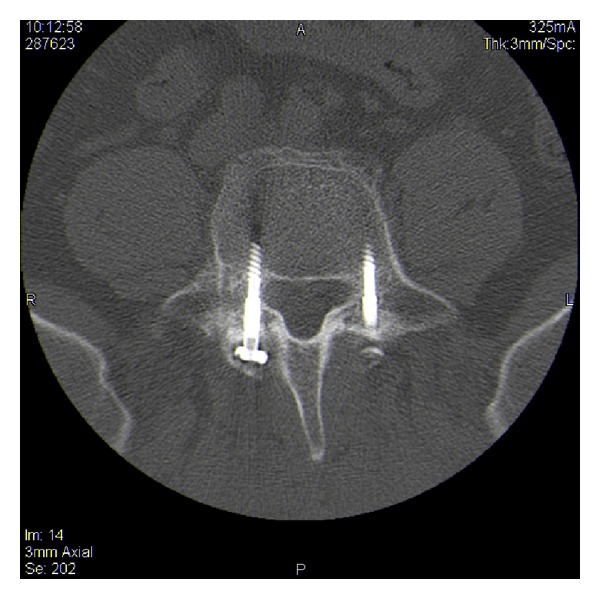
Bilateral lumbar fixation screws in the L5 vertebra.

**Figure 7:**
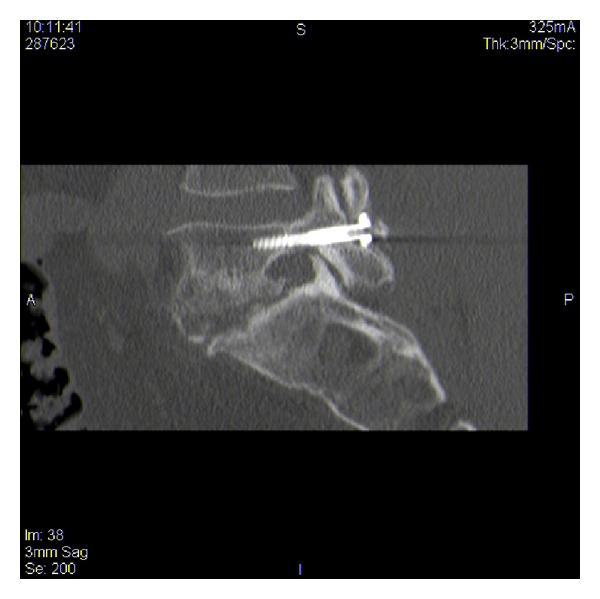
Sagittal view of right lumbar fixation screw in situ.

**Figure 8:**
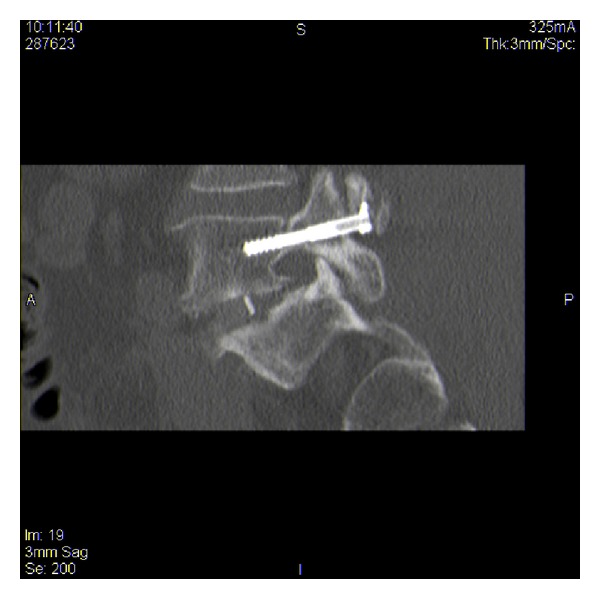
Sagittal view of left lumbar fixation screw in situ.

The percutaneous lumbar fixation screw used in this case (Figures 6, 7, and 8) is generally more robust than other small fragment screws. The percutaneous lumbar fixation screw is rigid and cannulated so it can be passed over a guide wire. It also has the advantage of being compressive by a mechanism that pulls the threaded portion locked in the bone towards the shank of the screw with its washer on the other side of the fracture gap so there is a direct compressive force rather than an applied force by rotation of the screw thread in the bone. Previous posterior fixation techniques often required a “figure of eight” wire around the transverse process to augment the screw. This was more invasive and failed if the transverse process was not strong enough to resist the compression and forced the wire cut through. 

## 4. Discussion

Percutaneous lumbar fixation is a minimally invasive option in the stabilization of complicated pedicle fractures. The problematic TDR had the potential to compromise the fixation of the fracture with an associated spondylolisthesis. Despite removal of the TDR and anterior fusion, this patient had a potentially unstable spine. Mathew et al. describe good result after salvage interbody fusion using a stand-alone anterior lumbar interbody fusion at L5/S1 level for a patient pars fracture and dislocated TDR but with no spondylolisthesis [4]. Another paper utilized a posterior approach L5/S1 fusion for pars defect (also without spondylolisthesis) with good effect [5].

Aryan et al. champion the use of percutaneous lumbar interbody fusion as a stand-alone procedure to secure isolated pars fracture with no TDR in situ. The fusion is via a safe paracoccygeal corridor, particularly when unfavorable anatomy precludes anterior fusion techniques [8]. Tender et al. identify that percutaneous screw fixation has lower operative morbidity and is useful in the correction of challenging lumbosacral spondylolisthesis, such as presentation in this patient [9]. The percutaneous system also spares the posterior muscles from detachment and provides rapid recovery [10].

A literature review comparing open versus percutaneous lumbar surgery does not demonstrate any benefit as yet [11]. The disadvantages of percutaneous surgery include a “loss of orientation, steep learning curve, increased radiation dose, and a reliance of technology” [11]. However, within this literature review, there are no large studies testing percutaneous lumbar spinal surgery. Much of the literature on percutaneous lumbar surgery is based on specific presentations whereby a minimally invasive approach is favorable. The adaptability of this percutaneous lumbar system to provide fixation was a key factor for why this device was used in this patient.

The patient in this case had an excellent and swift recovery following the percutaneous fixation and anterior fusion and has returned to playing golf. It is important to recognize which patients require an additional secure lumbar interbody fusion, but also those with abnormal anatomy and who will benefit from this less extensive approach.
